# The effect of an infra-acetabular screw for anatomically shaped three-dimensional plate or standard plate designs in acetabulum fractures: a biomechanical analysis

**DOI:** 10.1007/s00068-021-01805-x

**Published:** 2021-10-07

**Authors:** I. Graul, I. Marintschev, A. Pizanis, S. C. Herath, T. Pohlemann, T. Fritz

**Affiliations:** 1 Jena University Hospital, Department of Trauma, Hand and Reconstructive Surgery, Friedrich Schiller University Jena, Erlanger Allee 101, 07740 Jena, Germany; 2 Department of Orthopedics, Campus Eisenberg, University of Jena, Eisenberg, Germany; 3 Department for Trauma, Hand- and Reconstructive Surgery, Saarland University Medical Center, Kirrbergerstr. 100, 66421 Homburg, Germany; 4 BG Trauma Center, Eberhard Karls Universitaet Tuebingen, Tuebingen, Germany

**Keywords:** Acetabular fracture, Biomechanics, Infra-acetabular screw, Anatomically shaped plate, Suprapectineal

## Abstract

**Background:**

Various plate shapes and implant configurations are used for stabilization of acetabulum fractures via anterior approaches. Little is known about the biomechanical stability of a two-dimensionally shaped “conventional” plate (“J-Plate”—JP) in comparison to three-dimensionally shaped plate configurations (3DP). In addition, the augmentary effect of an infra-acetabular lag-screw (IACS) fixation for anterior column and posterior hemi-transverse acetabulum fractures has not been clarified in comparison of JP and 3DP constructs. This study analyzed the difference between the biomechanical stability of JP compared to 3DP and the role of an IACS in a standardized acetabular fracture model in a single-leg stance loading configuration.

**Methods:**

In an artificial bone substitute pelvis model (Synbone^©^ Malans, Switzerland), a typical and standardized fracture pattern (anterior column and posterior hemi-transverse) was created with osteotomy jigs. After anatomic reduction the stabilization was performed using JP or 3DP. Eight pelvises per group were axially loaded in a single-leg stance model up to 400 N. After the load cycle, an additional infra-acetabular screw was placed and the measurement repeated. Fragment displacement was recorded by an optical tracking system (Optitrack Prime 13®, Corvallis, USA).

**Results:**

In the pure placement, 3DP provided significantly superior stability when compared to JP. Augmentation of JP by IACS increased the stability significantly, up to the level of 3DP alone, whereas augmentation of the 3DP did not result in further increase of overall stability.

**Conclusion:**

The anatomically shaped plate alone provides a superior biomechanical stability in fixation of an anterior column and posterior hemi-transverse fracture model. In a JP fixation the augmentation by IACS provides similar strength as the anatomically shaped 3DP. By use of the anatomically shaped 3DP the need of a clinically risky application of IACS might be avoidable.

**Level of evidence:**

IV, Experimental study.

## Introduction

Open reduction and internal fixation with a plate osteosynthesis is considered to be the gold standard for the treatment of displaced acetabular fractures [1–3]. For this purpose, a multitude of different plate designs have been developed. Modern anatomically pre-shaped plates provide more possibilities to address displaced fractures, especially if the quadrilateral surface needs to be stabilized. Previous studies clearly showed that the development of post-traumatic osteoarthrosis is related to residual displacement and stability of fixation (“loss of reduction”) [2, 4–9]. However, even after anatomical reduction, loss of fixation can lead to secondary displacement and finally to hip joint failure resulting in total hip arthroplasty (THA) in up to 17% of the cases within 24 months after injury [1, 2, 10, 11]. Therefore, a reliable stable fixation after anatomical reduction is mandatory for long-term success.

Whereas in younger patients with good bone stock usually standard small fragment plates and screws provide sufficient stability [12], the rapidly increasing group of geriatric fracture patients require more sophisticated plate and screw designs and configurations to provide permanent stability over the course of healing. Various studies examined several implant shapes and screw configurations, e.g., Culemann et al. described a significant increase of fixation stability using the longest possible screws in the peri-articular region [13]. This was the basis of the concept of “peri-acetabular frame closure" elaborated in several subsequent studies [14–16]. Especially the infra-acetabular column screw (IACS) significantly augmented every plate construct [15, 16]. While biomechanically convincing, the clinical application of the IACS can be challenging due to the narrow infra-acetabular bone corridor with a not neglectable risk of intra-articular implant positioning [3, 17]. To improve the safety of implant positioning without compromising stability anatomically preformed 3D plate designs were developed and are in wide clinical application [18]. These are characterized by a direct fixation option of both columns via a buttress part supporting the quadrilateral surface. The additional buttress part with screw holes in the caudal region may take over the function of an infra-acetabular screw (IACS). The 3DP (SPP, Stryker, Duisburg, Germany) has an additional classical plate part with screw holes, which are located above the iliopectineal line. A further increase in fracture stability can be expected by placing the screws perpendicular to each other.

The aim of this biomechanical study was to evaluate stability after reduction and fixation of a frequent fracture acetabular fracture type (anterior column, posterior hemi-transverse) in comparison between a three-dimensional anatomically shaped plate and a standard iliopectineal plate. Additionally, the effect of additional fixation of each plate by an IACS was quantified.

## Methods

### Specimens

**Fig. 1:**
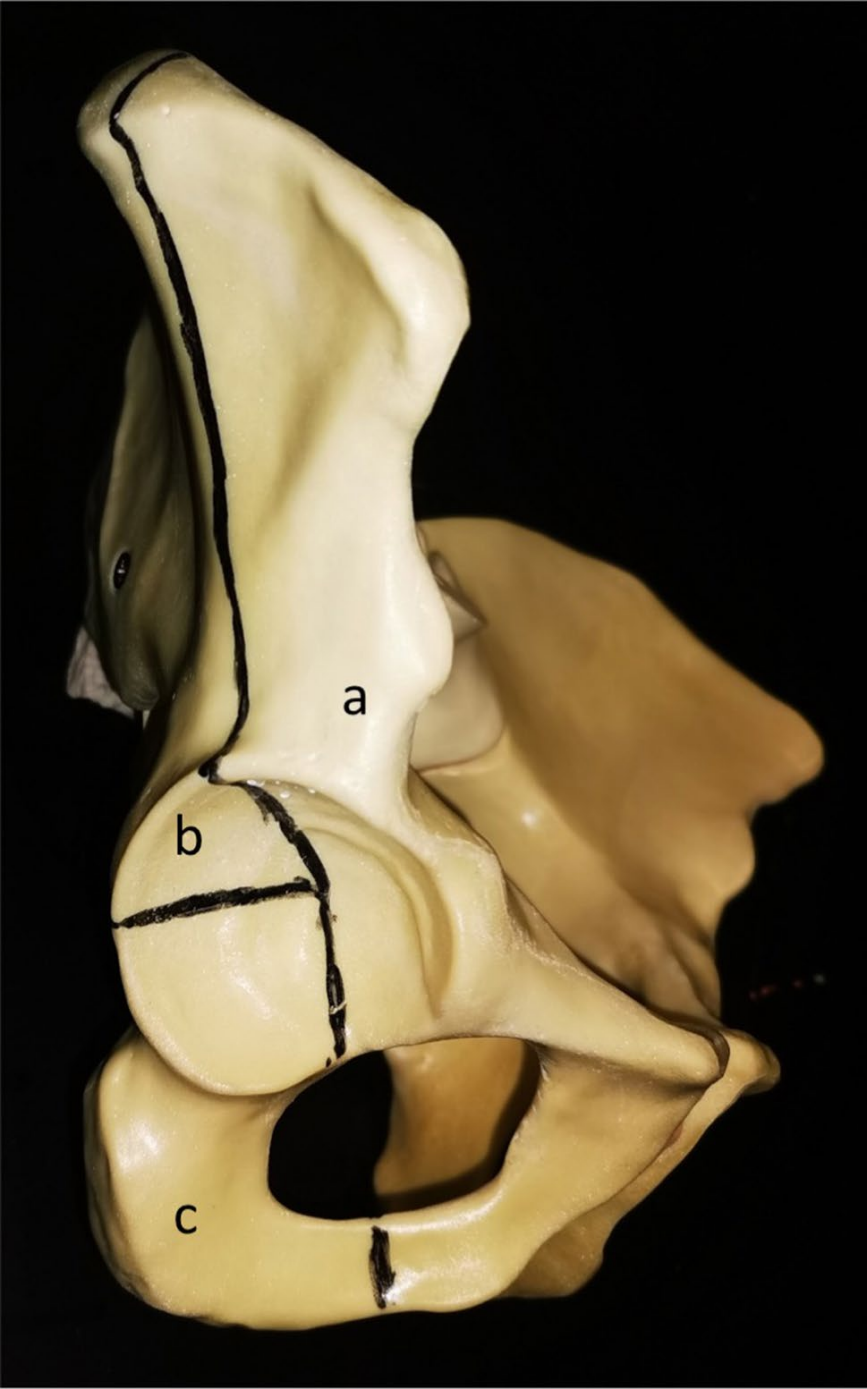
The template for the osteotomy of the acetabular fracture (anterior column and posterior hemi-transverse). a) Anterior column fragment, b) posterior column superior fragment, c) posterior column inferior fragment

**Fig. 2:**
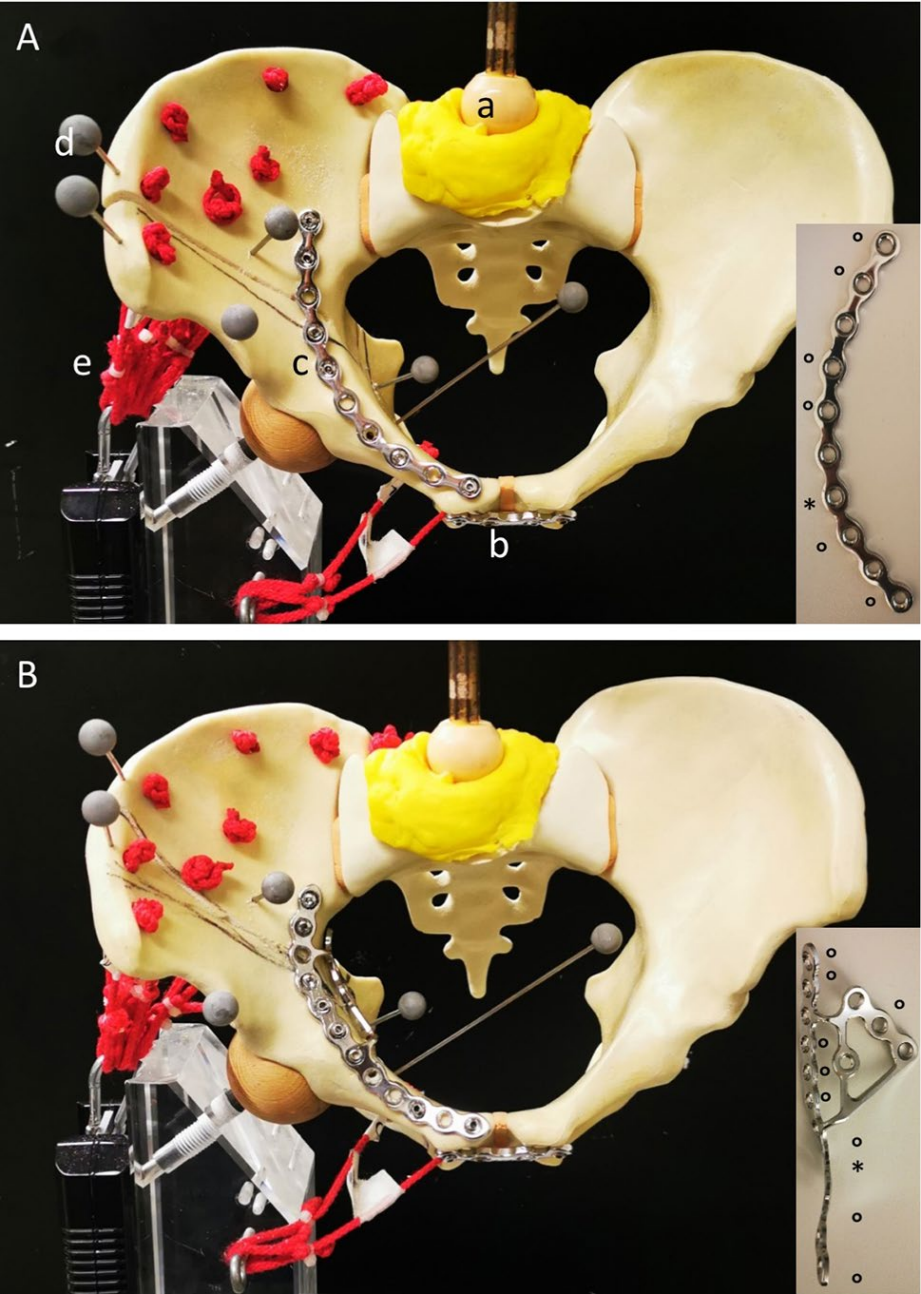
**A** Pelvis mounted in a single-leg stance with simulation of muscle traction (M. gluteus med. and M. quadratus fem.) and application of load through the sacrum. a) Axial loading via femoral head of a hip prosthesis, b) anterior plate stabilization c) JP stabilization, d optical tracking markers. **B** Pelvis mounted in a single-leg stance with 3DP stabilization. ° marks the screws placed for the stabilization of the fracture, * marks the plate hole for the IACS

Anatomical composite pelvises were used in this study (Model 4060®, Synbone, Malans, Switzerland). To simulate an acetabulum fracture (anterior column and posterior hemi-transverse, Fig. 1) a template for the osteotomy was created and used in all specimens. Due to the lack of ligament structures, the iliosacral joints were fixated using transsacral screws (Synthes, Oberdorf, Switzerland) on both sides and the symphysis was stabilized using a 4-hole interlocking symphysis plate (Synthes, Oberdorf, Switzerland) [13, 16, 19] (Fig. 2A, B).

### Sensors

To track 3D-Motion of the fracture fragments, an optical measuring system consisting of 4 cameras was used (Prime 13®, Optitrack, USA). The optical system detects with a resolution of 1280 × 1024 Pixel which allows an accuracy of 0.2 mm, as stated by the company. The system specific software (Motive 2.1®, Optitrack, USA) was used to process the measurements into a respective three-dimensional coordinate system. For evaluation, three main fragments were defined (“anterior column fragment”, “posterior column superior fragment” and “posterior column inferior fragment”). Each fragment was marked with 2 optical markers according to a template to achieve reproducible positioning. Mathematical calculations specified the relative movement of the main fragments in three spatial planes [11, 19]. The maximum fracture displacement was defined as the maximum fragment distance at loading. As preliminary tests showed a complete unstable fracture configuration without osteosynthesis. A comparative study without control group was conducted.

### Experimental set-up

In this study, we investigated the following 4 groups (*n* = 8). The pelvises in Group 1 and 3 were analyzed, then the additional IACS was placed in Groups 2 and 4.

Group 1: In this group, we stabilized the acetabulum fracture using the reconstruction JP, 10 holes curved, radius 88 mm (Synthes, Oberdorf, Switzerland) (Fig. 2A).

**Fig. 3:**
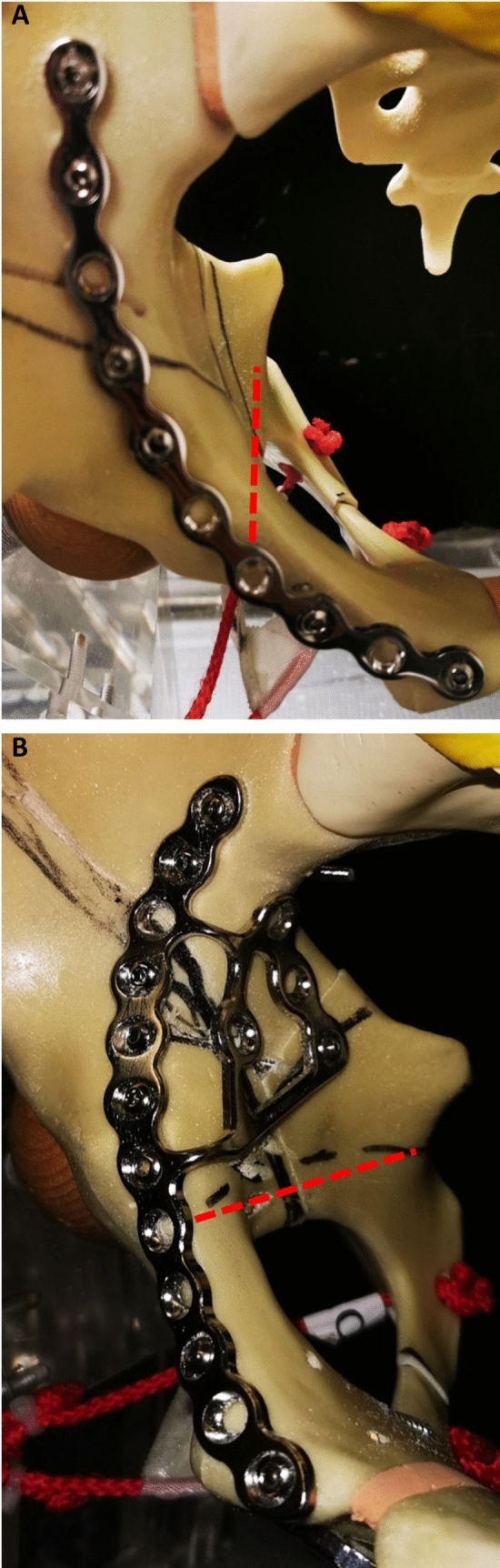
**A** The red line illustrates the corridor of the IACS for the JP. **B** The red line illustrates the corridor of the IACS for the 3DP

Group 2: In this group, an additional infra-acetabular screw (80 mm IACS, Synthes, Oberdorf, Switzerland) was placed at the already stabilized acetabulum fracture by the reconstruction JP, 10 holes curved, radius 88 mm (Synthes, Oberdorf, Switzerland) (Fig. 3A).

Group 3: In this group, we stabilized the acetabulum fracture using the anatomically pre-shaped suprapectineal plate 3DP (SPP 12 + 3 holes, Pelvic PRO System, Stryker, Duisburg, Germany) (Fig. 2B).

Group 4: In this group, an additional infra-acetabular screw (80 mm IACS, Synthes, Oberdorf, Switzerland) was placed at the already stabilized acetabulum fracture by the pre-shaped suprapectineal plate 3DP (SPP 12 + 3 holes, Pelvic PRO System, Stryker, Duisburg, Germany) (Fig. 3B).

To allow a controlled load distribution of the affected half of the pelvis, all pelvises were tested by a simulated single-leg stance model as shown in previous studies [14, 20–22]. The 6 Optical markers (Optitrack, USA) were added to the fracture fragments (2 for each fragment) as recommended by the manufacturer.

Then, each pelvis was mounted into a universal testing (INSTRON E10000, Instron, Norwood, USA). To simulate a single-leg stance, a custom-built mount was used as in previous studies [14, 20]. High-strength nylon cords were used, to simulate the muscle tracts of the gluteal and quadratus muscles, which simultaneously allowed free movement of the pelvis in all three spatial coordinates. All pelvises were axially loaded using a ceramic hip prosthesis head (28 mm, Zimmer Inc., Warsaw, Indiana, USA) articulating at 45° to the proximal sacrum in a custom-made fitting cement cup (Technovit 3040®, Heraeus Kulzer GmbH, Wehrheim, Germany) on the first sacral vertebra.

The pelvis models were positioned with a preload of 50 Newton, whereby a 25° inclination and 15° abduction were set, which reflects the vector of the maximum load during a gait cycle shortly before foot lifting. The respective maximum load and relief plateau was held for 10 s each. The first 2 loads were defined as setting cycles. Then the fracture displacement location was measured during the following 5 cycles. The force was reduced to the 50 N initial force and increased by 50 N–400 N to prevent complete destruction of the specimen. When a failure of the osteosynthesis was detected, the testing was stopped immediately. The failure of the osteosynthesis was defined as fracture displacement of 2 mm, implant or pelvic fracture.

### Data acquisition, processing and statistical analysis

The spatial instability and movement were acquired by an optical measurement system (Prime 13®, Optitrack, USA). The marker pairs were mounted at standard positions in each pelvis. The analysis was performed using the software provided by the manufacturer (Motive 2.1®, Optitrack, USA).

Statistical evaluation of the individual groups was performed with the SPSS software (version 22.0, Chicago, IL) and SigmaPlot (SigmaPlot 13.0; Systat Software Inc., San José, USA), using Wilcoxon test. For continuous variables mean values and standard deviations were calculated. Results were considered statistically significant if the corresponding *p*-value was < 0.05.

## Results

The 3DP showed an exact fit on the artificial bone model and did not need any additional bending. The JP had to be contoured preoperatively. Change in distance between the three fragments and rotation of the fragments in space were evaluated. Up to the maximum loading level of 400 N no failure was observed.

The failure of plate osteosynthesis in anterior column and posterior hemi-transverse fracture was most evident in the displacement of the posterior column superior fragment.

Although multiple distances and angles were evaluated, a representative marker pair is presented as an example in the results for clarity. The mean values and standard deviations of the fragments for the representative marker pair per fragment are shown in Table 1.

**Table 1:** Distance in mm (given in mean value ± standard deviation) and angle in degrees (given in mean value ± standard deviation)

	JP		3DP	
	Without infra-acetabular screw	With infra-acetabular screw	Without infra-acetabular screw	With infra-acetabular screw
Anterior column fragment–posterior column inferior fragment in [mm]	1.33 ± 1.31	0.89 ± 0.59	0.72 ± 0.44	0.63 ± 0.43
Anterior column fragment–posterior column superior fragment in [mm]	0.81 ± 1.15	0.45 ± 0.84	0.49 ± 0.61	0.26 ± 0.47
Posterior column inferior fragment–posterior column superior fragment in [mm]	2.89 ± 1.61	1.99 ± 0.59	2.05 ± 1.11	2.17 ± 1.34
Angle anterior column fragment posterior column inferior fragment in [°]	0.22 ± 0.17	0.21 ± 0.16	0.12 ± 0.09	0.20 ± 0.16
Angle anterior column fragment–posterior column superior fragment in [°]	0.30 ± 0.23	0.19 ± 0.17	0.45 ± 0.47	0.23 ± 0.20
Angle posterior column inferior fragment–posterior column superior fragment in [°]	2.17 ± 1.29	1.50 ± 0.41	1.12 ± 0.77	1.31 ± 0.76

**Fig. 4:**
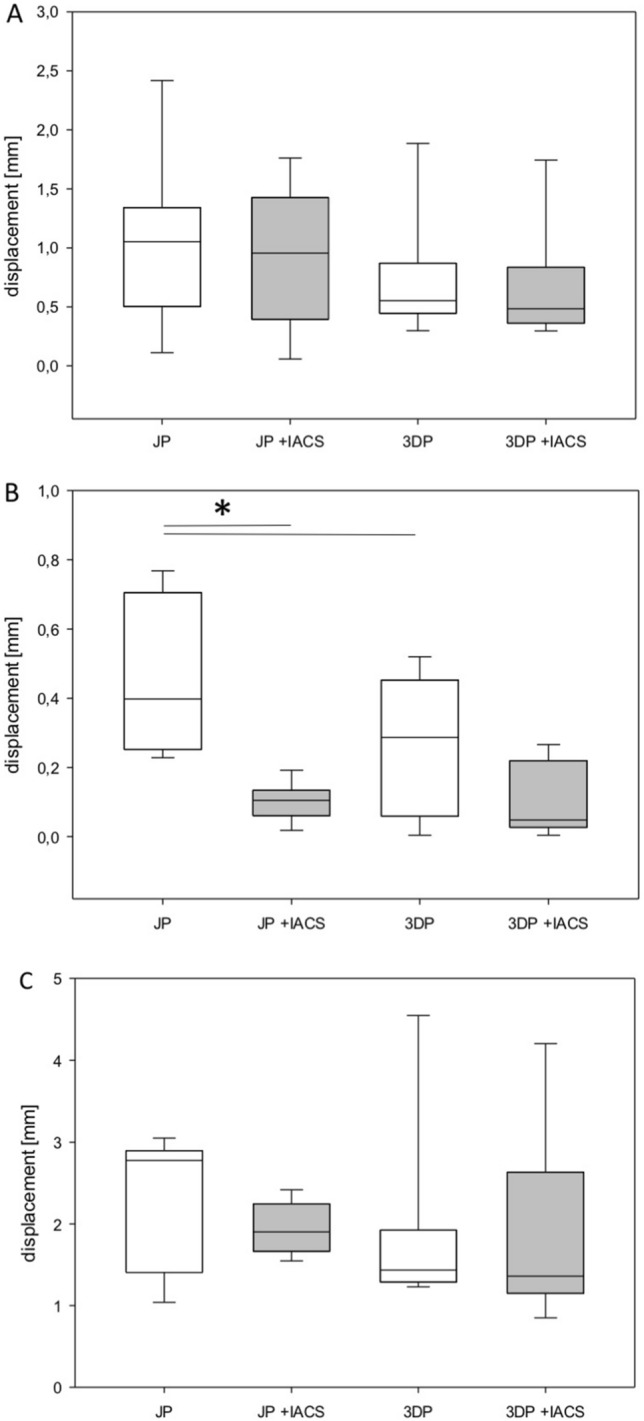
Fragment displacement with three-dimensional anatomically pre-shaped plate and JP with and without IACS in mm. **A** Displacement between the anterior column fragment and the posterior column inferior fragment. **B** Displacement between the anterior column fragment and the posterior column superior fragment. **C** Displacement between the posterior column superior fragment and the posterior column inferior fragment. * *p* < 0.05 vs. JP, # *p* < 0.05 vs. 3DP

**Fig. 5:**
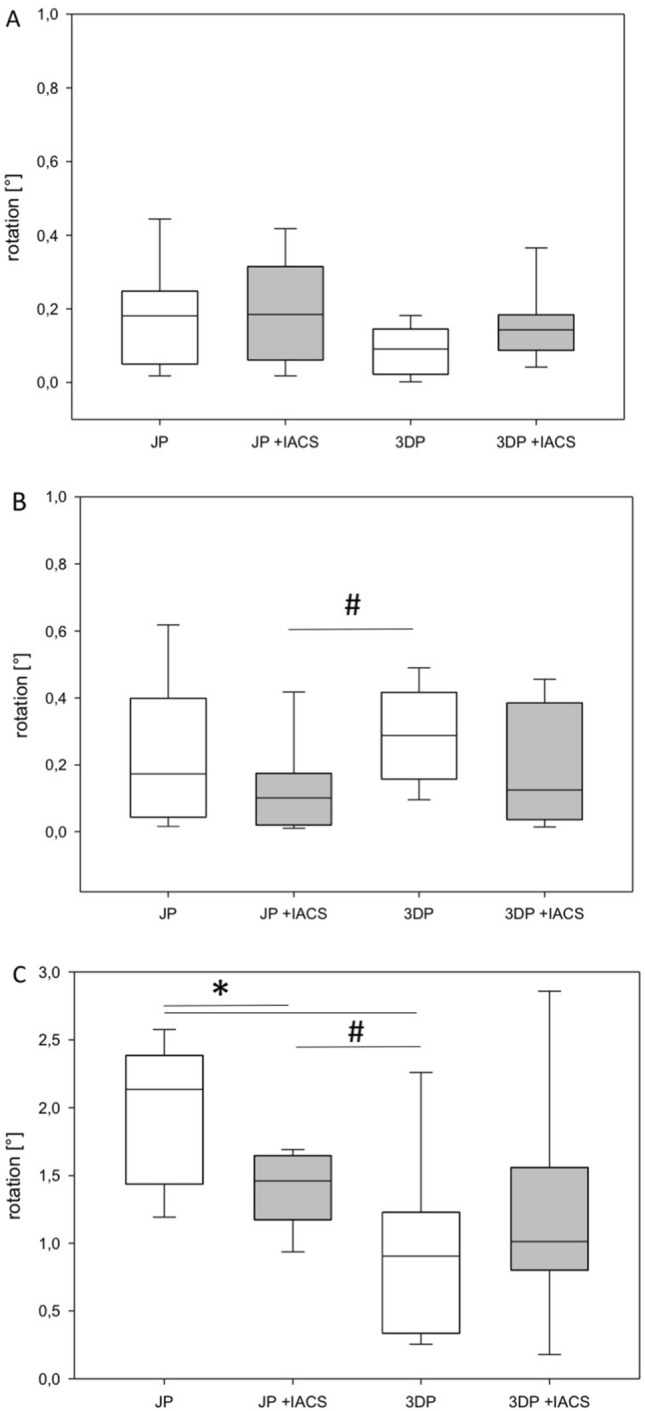
Fragment rotation with three-dimensional anatomically pre-shaped plate and JP with and without IACS. **A** Rotation between anterior column fragment and posterior column inferior fragment. **B** Rotation between posterior column superior fragment and anterior column fragment. **C** Rotation between posterior column superior fragment and posterior column inferior fragment. * *p* < 0.05 vs. JP, # *p* < 0.05 vs. 3DP

In comparison to the JP without IACS, the 3DP without IACS showed a significantly higher fixation strength in counteracting the displacement tendency in terms of gap increase of the anterior to the posterior superior fragment (*p* < 0.05 anterior column vs. posterior column superior fragment) (Fig. 4B). There was no difference in the displacement of the anterior to the posterior inferior fragment (Fig. 4A) or posterior superior fragment to posterior inferior fragment (Fig. 4C). Furthermore, the rotation stability was significantly lower for the JP without IACS in comparison to the 3DP without IACS (*p* < 0.05 posterior column superior fragment vs. posterior column inferior fragment) (Fig. 5C). There was no difference in the rotation stability of the anterior to the posterior inferior fragment (Fig. 5A) and anterior to the posterior inferior fragment (Fig. 5B).

Looking at the JP alone, additive placement of an IACS in the infra-acetabular corridor results in significantly greater stability of the anterior column fragment and the posterior column superior fragment (*p* < 0.05) (Fig. 4B). Occupation of the infra-acetabular corridor by an IACS did not result in increased stability of the anterior to the posterior inferior fragment (Fig. 4A) or the posterior superior fragment to the posterior inferior fragment (Fig. 4C). The rotational stability posterior column superior to the posterior column inferior fragment increased significantly after the IACS was placed (*p* < 0.05; Fig. 5C). No significant effects were measured for the other fragments (Fig. 5A, B).

Looking at the 3DP alone, no significant effect on the fixation strength and rotational stability of the fragments was observed when an additive IACS was placed through the infra-acetabular corridor (Figs. 4 & 5).

Comparing the 3DP without IACS vs. the JP with an additive IACS, the 3DP had less effect on the rotational stability of the anterior to the posterior column superior fragment (*p* < 0.05) (Fig. 5B). However, rotational stability of the posterior column inferior fragment was increased by fixation with 3DP (*p* < 0.05) (Fig. 5C).

## Discussion

This study compared the fixation strength of the JP with the anatomically shaped suprapectineal plate and evaluated the value of an additional infra-acetabular screw.

Due to the relevance, an anterior column and posterior hemi-transverse fracture was selected as fracture model. This fracture type represents 17.1% of all acetabular fractures seen in Germany and is constantly increasing over the last 15 years [12, 23, 24]. It is a typical fracture pattern of the old age, in line with the accident mechanism and bone quality [13, 25]. In the German Pelvic Registry (2002–2017), 50.5% of the patients with acetabular fracture were older than 60 years [26].

Previous studies showed that displaced acetabular fractures involving the quadrilateral surface often result in post-traumatic osteoarthrosis based on malreduction and secondary loss of fixation [7, 8, 13, 27, 28]. To address this problem, previous studies analyzed alternative fixation methods of the quadrilateral surface. Therefore acetabular plates with wire or cable cerclages [20, 29], buttress plates [30, 31] or special quadrilateral surface plates have been described [27, 30, 32]. The introduction of anatomically pre-shaped plates gained an increasing popularity in acetabular surgery, with promising results so far [18].

However, the optimal fixation to achieve high biomechanical stability of the acetabulum, especially with limited bone quality, is still discussed. Placing peri-articular screws, as described by Letournel and Judet, is an option to enhance the stability of implants in acetabular fracture surgery [33]. Culemann et al. [14] proposed a simple modification to connect both osseus columns with an infra-acetabular screw. Marintschev et al. [16] showed that the use of locking screw systems did not improve the stability for common pelvic plate systems. In all tested plate systems supplemental placement of the IACS through the plate significantly improved biomechanical stability in fractures of the anterior column. The infra-acetabular column screw can be used for isolated screw fixation or as an additive to plate fixation for anterior column fractures. The additional gain in fixation strength has been demonstrated by Gras et al. on artificial bone models [15].

In this study, the biomechanical properties of the anatomically 3DP in combination with the IACS were evaluated and compared to standard JP, also in combination with an IACS.

For biomechanical testing, standardized fractures were generated by osteotomies on artificial synthetic pelvis models, as described in previous studies [21, 34]. This model has limitations due to missing ligaments, however artificial pelvises allow a very standardized sample group which overcomes the nearly uncountable variations in bone quality seen in human anatomical specimens [22, 25, 35]. To analyze the movement and stability of the fracture fragments, a single-leg stance model was used [14]. This model acts as a “worst case scenario”, concentrating all forces during loading into the region of interest [14]. The axial loading force was limited to 400 N, as described in the literature to ensure relevant loading comparable to partial weight bearing, minimizing fatal displacements and therefore allow a secondary augmentation by IACS [36].

Our results show that the additional screw row of the 3DP in the area of the quadrilateral surfaces increases the support in this area in comparison with the conventional JP. These results are comparable to the results of Schäffler et al. although in their study the usage of an IACS was not investigated [21].

As expected the additional IACS increased the stability of the fragments significantly for the JP group as described in the literature [14, 16], whereas in the 3DP group no further stabilizing effect could be detected. However, a slight decrease of the rotational stability within the 3DP + IACS group was detected for the posterior column superior and inferior fragment, which could be explained by wedging of the fragments due to additive screw positioning. Still, no significant difference was detected. Thus, the conclusion can be drawn, that the intrinsic stability of a “3D shaped implant” can provide a secure connection between the anterior and posterior column, even without formal lag-screw fixation; whereas in conventional plate designs, the IACS remains an elementary element of a stable fixation construct. Despite studies to simplify the search for the infra-acetabular corridor [3], Gras et al. showed that in 6–7%, the anatomical corridor does not allow the placement of an IACS [17]. So, the IACS is still challenging, time-consuming and may be associated with the complication of intra-articular screw misplacement.

## Conclusion

Anatomically shaped plate systems are valuable additions to the implant section used for acetabulum fractures which might facilitate stabilization. Conventional plate/screw combinations still have their place, even for fixation in geriatric patients, provide adequate stability but certainly require an advanced skill level of the responsible surgeon. The 3D preformed plate might be a safe alternative providing adequate stability in cases where placement of an IACS is not possible or too surgically demanding.
